# FLT4 activation promotes acute lymphoid leukemia survival through stabilization of MDM2/MDMX and inactivation of p53

**DOI:** 10.1038/s41389-025-00552-7

**Published:** 2025-05-02

**Authors:** Djazia Haferssas, Marion Dubuissez, Jonatan Barrera-Chimal, Clémence Messmer, El Bachir Affar, Bruno Larrivée, Xue-Song Liu, Casimiro Gerarduzzi

**Affiliations:** 1 Département de Pharmacologie et Physiologie, Faculté de Médecine, Université de Montréal, Montréal, QC Canada; 2 Centre de Recherche de l’Hôpital Maisonneuve-Rosemont, Centre Affilié à l’Université de Montréal, Montréal, QC Canada; 3 Département de Microbiologie et Immunologie, Faculté de médecine, Université de Montréal, Montréal, QC Canada; 4 School of Life Science and Technology, ShanghaiTech University, Shanghai, China; 5 Département de Médecine, Faculté de Médecine, Université de Montréal, Montréal, QC Canada

**Keywords:** Acute lymphocytic leukaemia, Stress signalling

## Abstract

Aberrant Receptor Tyrosine Kinase (RTK) signaling allows cancer cells to modulate survival, proliferation, and death, leading to tumorigenesis and chemoresistance. In leukemia, the RTK FMS-Related Tyrosine Kinase 4 (FLT4) (also known as VEGFR3, Vascular Endothelial Growth Factor Receptor- 3) is deregulated and correlates with cancer progression. However, the underlying consequences of its deregulation remain to be determined. Moreover, chemotherapy treatment requires that cancer cells retain a wild-type p53 to respond to DNA damage by tumor-suppressing activities, i.e. apoptosis. p53 activity is predominantly limited by its two major negative regulators, MDM2 and MDMX, which inactivate p53 by promoting its degradation and/or cytoplasmic localization. In this study, we have shown that activation of FLT4 by either overexpression or binding of its ligand, VEGFC, increases MDM2/MDMX stability, inactivates p53, and leads to resistance to DNA-damaging therapies. Moreover, we found that MDMX Ser-314 phosphorylation, a consensus sequence of CDK4/6, increases MDMX stability, which subsequently affects MDM2 and p53 degradation and could be reversed by the CDK4/6 inhibitor Palbociclib. More importantly, leukemic cells treated with Palbociclib were more susceptible to DNA-damaging induction of apoptosis and had reduced cell proliferation. Leukemic cells overexpressing FLT4 displayed accelerated proliferation when injected into NOD-SCID mice as compared to wild-type cells. Altogether, our research proposes an innovative way to reactivate p53 in leukemia through the pharmacological inhibition of FLT4 signaling, which could serve as a potential treatment option.

**Figure:**
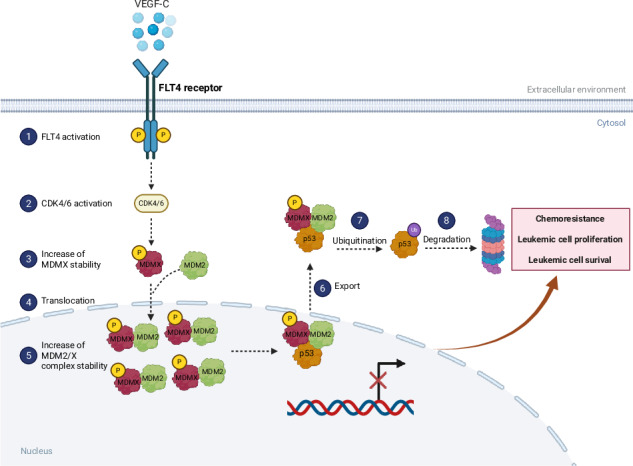
Schematic representation of FLT4-mediated MDM2/MDMX complex stabilization and suppression of p53 activity. VEGFC triggers FLT4 activation, leading to CDK4/6 activation, which phosphorylates MDMX on Ser-314. As a result, MDMX levels increase and bind to MDM2, stabilizing the MDM2/MDMX complex. This complex binds to p53, facilitating its suppression by reducing its transcriptional activity or enhancing its export to the cytoplasm for proteasomal degradation. Consequently, p53 inactivation promotes their survival, proliferation, and resistance to chemotherapy-induced apoptosis. *The figure was created in BioRender.com*.

Schematic representation of FLT4-mediated MDM2/MDMX complex stabilization and suppression of p53 activity. VEGFC triggers FLT4 activation, leading to CDK4/6 activation, which phosphorylates MDMX on Ser-314. As a result, MDMX levels increase and bind to MDM2, stabilizing the MDM2/MDMX complex. This complex binds to p53, facilitating its suppression by reducing its transcriptional activity or enhancing its export to the cytoplasm for proteasomal degradation. Consequently, p53 inactivation promotes their survival, proliferation, and resistance to chemotherapy-induced apoptosis. *The figure was created in BioRender.com*.

## Introduction

Acute leukemia is a group of malignant disorders characterized by aberrant proliferation of hematopoietic stem cells and progenitors in the blood and bone marrow [1]. Acute Lymphoid leukemia (ALL) is the most common cancer in children [2]. Although chemotherapy treatments have achieved up to 90% remission [2, 3], survival remains poor for 10% of these patients due to chemotherapy resistance [4, 5]. Furthermore, effective doses cause severe late effects of toxicity [6–8]. Therefore, a better understanding of these DNA-damaging therapies would permit precise and safer treatments for leukemic cell death.

Most chemotherapies are DNA-damaging therapies [9] that primarily rely on activating the tumor suppressor p53. Indeed, p53 has multiple biological functions associated with tumor suppression, such as cell cycle arrest, DNA repair, and apoptosis [10, 11]. To avoid p53 lethality, two major negative regulators, MDM2 (murine double minute protein 2) and its homologous MDMX (murine double minute 4 homolog MDM4) [12], control p53 activity by either the complex MDM2/MDMX binding p53 to prevent its transcriptional activity or MDM2 ubiquitinating p53 for proteasomal degradation [13, 14]. p53 is constantly expressed with a short half-life in normal conditions, thus, it is continually degraded by MDM2/MDMX to be maintained at low levels. In response to DNA damage, p53 rapidly uncouples from the MDM2/MDMX complex allowing its stabilization and activation of tumor-suppressing genes [13].

Due to its essential role in preventing tumorigenesis, p53 is the most frequently mutated gene in human cancers, allowing tumor cell proliferation. Over 50% of solid tumors have p53 mutations [15]. However, p53 mutations occur in less than 10% of leukemic patients [16–19], thus, inactivation rather than mutation of p53 likely plays a role in leukemogenesis, suggesting the potential of p53 reactivation to improve treatments. In ALL, MDM2 and MDMX are overexpressed [20–27], representing attractive targets to restore p53 functions [20]. Aberrant activation from Receptor Tyrosine Kinases (RTKs) allows cancer cells to abnormally control the cellular processes of survival, proliferation, and cell death, leading to tumorigenesis and drug resistance [28–30]. Several findings have supported the role of the RTK FMS-Related Tyrosine Kinase 4, FLT4, also called VEGFR3 (Vascular Endothelial Growth Factor Receptor- 3) and its ligand, VEGFC (Vascular Endothelial Growth Factor-C), in cancer progression [30, 31]. In pediatric ALL, VEGFC protein was detected in 27% of patients, which was significantly associated with ALL treatment failure [31]. VEGFC signaling has also been shown to play an important role in decreased drug response and chemotherapy resistance in acute leukemia [28–30]. Furthermore, in vitro VEGFC treatment increased leukemic cell survival and proliferation [29, 30], and protected against apoptosis [29, 30]. Although there is a strong association between FLT4 and leukemia, they have been understudied, and consequently, the mechanism by which FLT4 may be implicated in leukemia and therapy resistance is unknown.

Given that ALL patients have a wild-type p53 with often elevated levels of FLT4/VEGFC, and resistance to DNA damage-induced apoptosis, we investigated whether the modulation of MDM2/MDMX complex sits at the interface of FLT4 and p53 activity. The present study shows that FLT4 leads to increased MDM2/MDMX complex stability, potentially through CDK4/6 and p53 inactivation. We have also shown that reactivating p53 in FLT4-treated leukemic cells through CDK4/6 inhibition increased their responsiveness to genotoxic drugs promoting their apoptosis and thus providing a mechanism of combination therapy.

## Material and methods

### Cell culture

The Human Embryonic Kidney cell line (HEK293T) and the Human Bone Osteosarcoma Epithelial cell line (U2OS) from American Type Culture Collection (ATCC) were cultured in Dulbecco’s Modified Eagle’s Medium (DMEM, Gibco) supplemented with 10% of Fetal Bovine Serum (FBS, Gibco) and Penicillin/Streptomycin at 37 °C in a humidified atmosphere of 5% CO_2_. Mycoplasma contamination was assessed frequently by PCR following the previously described methods [32, 33].

For genotoxic treatment, U2OS cells were treated with 10 μg/mL of 5-Fluorouracil (5-FU) at various time points.

The human precursor B-ALL cell line (Reh) from ATCC was cultured in Roswell Park Memorial Institute media (RPMI 1640, Gibco) supplemented with 10% of FBS at 37 °C in a humidified atmosphere of 5% CO_2_.

In each experimental condition, Reh cells were starved overnight and treated with 100 ng/mL of recombinant human rhVEGFC (R&D Systems) for FLT4 activation or with 5 μM of FLT4 inhibitor (MAZ51, Sigma Aldrich) at different time points. For genotoxic treatments, cells were treated with either 0.25 μM of Etoposide (Sigma-Aldrich) or 50 nM Doxorubicin (Sigma-Aldrich). For Cycloheximide (CHX) treatment, cells were pre-treated with 100 ng/mL of rhVEGFC for 3 h and then treated with 25 µg/mL of CHX at different time points. For combined treatment with CDK4/6 inhibitors, cells were pre-treated with Palbocilib (1 μM, Selleckchem) or Ribociclib (5 μM, Selleckchem) for 24 h, followed by Doxorubicin (50 nM). All in vitro experiments were performed in triplicate.

### Transient transfections and transductions

Transient transfections were carried out using Lipofectamine 2000 (Invitrogen) following the manufacturer’s protocol. Expression plasmids, including pCMV-Myc-MDM2, pCMV-MDM2 C464A mutant, pcDNA3-FLT4, and pcDNA3-Flag-MDMX, were obtained from Addgene (#16441, #12086, #119230, and previously described [34], respectively). The pcDNA3-Flag-MDMX S314A mutant was generated using the Quick Change II XL site-directed mutagenesis kit (Agilent Technologies) with the following primers: 5’GTACTGAATGCAAGAAATTTAACGCTCCAAGCAAGAGGTACTG3’ and 3’CAGTACCTCTTGCTTGGAGCGTTAAATTTCTTGCATTCAGTAC5’, according to the manufacturer’s instructions. Lentiviral transduction was performed in two rounds: initially, HEK293T cells were transfected with plasmids for lentivirus production using (3.74 μg psPAX2 + 1.25 μg pMD2.G) and 6 μg of dTomato-luciferase lentiviral vector, pUltra-Chili-Luc, all purchased from Addgene (#12260, #12259 and #48688 respectively). After 48 h, the supernatant containing viral particles was collected and filtered. Reh cells were infected with the collected supernatant supplemented with Polybrene (4–8 μg/mL) and sorted by flow cytometry based on dTomato expression. The second round of transduction was performed using pHAGE-GFP vectors or pHAGE-FLT4-GFP obtained from Addgene (#106281 and #116745, respectively) and GFP-positive cells were sorted and maintained in culture.

### Colony forming assay of Reh cells

For each condition, 500 Reh cells were resuspended in completed media and seeded in 1 mL of 1.2% methylcellulose (#4230, Stem Cell). Cells were plated in four replicates onto 35 mm^2^ tissue culture dishes and incubated in a humidified atmosphere at 37 °C and 5% CO_2_. After two weeks of culture, colonies consisting of at least 50 cells were counted using an inverted microscope.

### Growth curve analysis of Reh cells

For each condition, 100,000 Reh cells were seeded in a six-well plate in 3 mL of complete media and treated with 1 μM of CDK4/6 inhibitor (Palbociclib, Selleckchem). Cells were counted every 24 h for five consecutive days.

### Whole-cell extract preparation and Western Blot

Total protein extracts were obtained using RIPA supplemented with protease and phosphatase inhibitors (Roche), followed by homogenization and centrifugation. Protein concentration was determined with the Pierce BCA protein assay kit (Thermo Scientific), and proteins were denatured in Laemmli buffer. SDS-PAGE separated 15–20 μg of proteins and transferred onto PVDF membranes. After blocking, membranes were incubated overnight at 4 °C with primary antibodies: anti-MDM2 (86934S, Cell Signaling, 1:1000), anti-MDMX (3807946, Millipore, 1:1000), anti-FLT4 (3200622, Millipore, 1:1000), anti-phospho-Tyrosine (9416S, Cell Signaling, 1:1000), anti-p53 (Sc-126, Santa Cruz, 1:1000), anti-AKT (#9272, Cell Signaling, 1:1000), anti-phospho-AKT (#9271T, Cell Signaling, 1:1000), anti-p38 (#9212, Cell Signaling, 1:1000), anti-phospho-p38 (#9211, Cell Signaling, 1:1000), anti-Caspase 3 (#9662, Cell Signaling, 1:1000) and anti-GAPDH (9485, Abcam, 1:1000). Following primary antibody incubation, membranes were washed with TBS-T and incubated with HRP-conjugated secondary antibodies for 1 h at room temperature. Bands were visualized using Clarity Max ECL Western Substrate, and densitometry analysis was performed with the ImageQuant LAS-4000 system and FUJIFILM MultiGauge V3.0.

### MDM2 or MDMX immunoprecipitation

Following 24 h of HEK293T transfection, cells were lysed in IP buffer (Tris 1 M, pH=8, NaCl 5 M, EDTA 0.5 M, Nonidet P-40, 0.5%) with protease and phosphatase inhibitors (Roche) at 4 °C. Lysates were homogenized, then centrifuged at 13.000 × *g* at 4 °C, and protein was quantified using the Pierce BCA assay kit (Thermo Scientific). 1 mg of proteins were incubated with 50 μL Myc antibody-conjugated beads (Sigma- Aldrich) or Flag antibody-conjugated beads (Sigma- Aldrich) overnight at 4 °C. After centrifugation, the supernatant was removed, and beads were washed thrice with IP buffer, then denatured for 10 min at 95 °C. IP products were analyzed by Western Blot.

### Flow cytometry analysis for apoptosis

Reh cells were seeded until 80% confluency and treated with a gradient of genotoxic drugs (Doxorubicin or Etoposide). After 24 h (Etoposide) or 48 h (Doxorubicin), cells were washed three times with PBS and incubated at room temperature in Annexin V binding buffer (#42200, Biolegend,) containing APC-conjugated Annexin V antibody (#640919, Biolegend, 1:1000). After 15 min, Annexin V binding buffer containing DAPI (#422801, Biolegend, 1:500) was added. Apoptosis was immediately measured by Flow cytometry using BD LSR Fortessa. Data analysis was performed using FlowJo Software.

### FireflyDual luciferase assay

U2OS cells were transfected either with FLT4 expression vector alone or in combination with MDM2 and MDMX expression vectors as indicated, alongside with firefly luciferase reporter plasmids (pGL3-Empty, pg13-p53 binding sites, pg13-mdm2 or pGL3-p21) as previously described [35]. After 36 h of transfection, cells were analyzed using the Dual-luciferase Assay kit (Promega) according to the manufacturer’s instructions. Renilla luciferase reporter plasmid under the control of the β- globin [35] was used to normalize the data.

### Immunofluorescence analysis for MDM2/MDMX complex

After 24 h of transfection, cells were fixed with 4% paraformaldehyde and permeabilized in 0.1% Triton-X-100/phosphate-buffered saline. Coverslips were incubated with primary antibodies against MDM2 (86934S, Cell Signaling, 1:200) and MDMX (3807946, Millipore, 1:200) diluted in 1% BSA/PBS overnight. After washing, coverslips were incubated with secondary antibodies (donkey anti-rabbit AF-647, #711-605-152, Jackson ImmunoResearch, 1:200, and donkey anti-mouse Cy-3, #715-165-150, Jackson ImmunoResearch, 1:200) diluted in 1% BSA/PBS for 1 h. Coverslips were washed and mounted with DAPI. Image acquisition was performed with a Zeiss LSM880 multi-photon microscope, and data were analyzed using Zeiss Blue software.

### Tumor xenograft model

Male and female NOD SCID gamma mice, aged between 8 and 12 weeks, were used (*n* = 11–12). Mice were housed in a pathogen-free environment within the Maisonneuve-Rosemont Hospital Research Center animal facility and fed Harlan Teklad rodent diet (#2018 Envigo, Qc, CAN) and water ad libitum. All experiments were conducted according to the Canadian Council on Animal Care guidelines for the care and use of laboratory animals, and under the supervision and approval of our local animal care committee, Comité de Protection des animaux du CIUSSS de l’Est- -de-l’île-de-Montréal (approved protocol #2024-3409). Mice were randomly selected to receive 500,000 Reh cells transduced with luciferase, and vEmpty or vFLT4 via tail vein injections. Mice received intraperitoneal D-luciferin (Revvity, 150 mg/kg), were anesthetized with Isoflurane, and imaged on an in vivo imaging system (IVIS). Images were analyzed using Living Image software. On day 21, mice were euthanized, and lung imaging was performed after PBS wash. The investigator who performed the cell injections was blinded to the type of cells injected. For the group assignments, data collection and analysis, the investigators were not blinded, as the same team members who conducted the experiments also performed the data analysis.

### Statistical analysis

The results are expressed as mean ± standard error. Statistical significance for multiple comparisons was calculated using ANOVA, while for two-group comparisons was calculated by a Student *t* test. *P* value < 0.05 was considered statistically significant. Statistical analyses and graphical representation were performed using GraphPad Prism v6.07.

## Results

### FLT4 overexpression negatively regulates p53 by promoting MDM2/MDMX complex

**Fig. 1:**
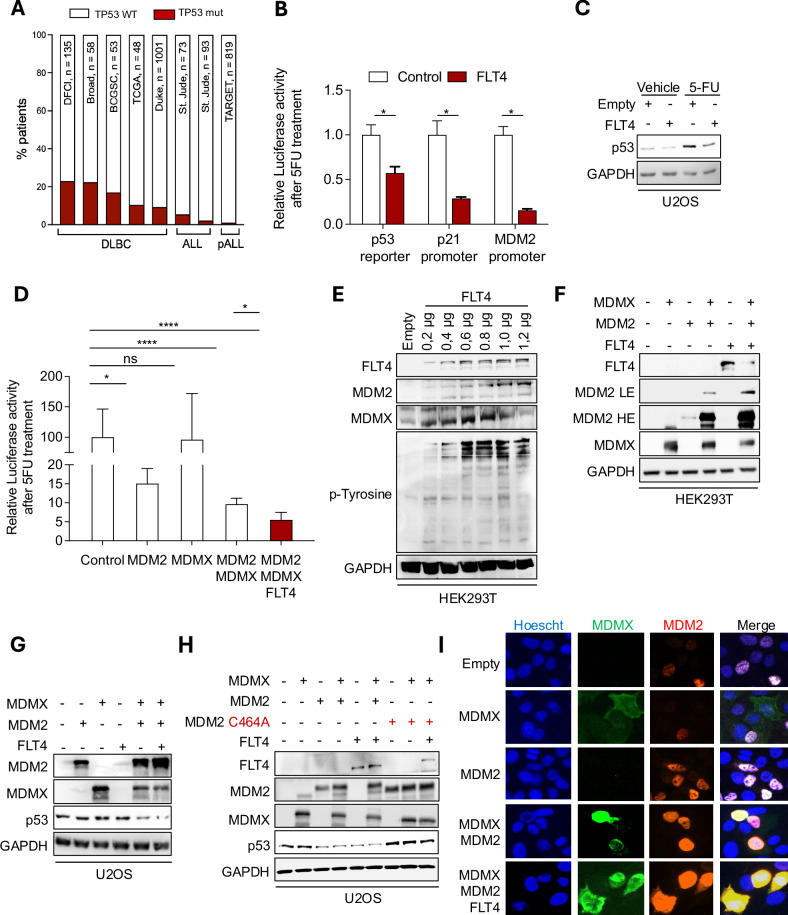
FLT4 overexpression negatively regulates p53 expression and localization. **A** The frequency of p53 mutation in Diffuse Large B Cell lymphoma (DLBC) patients *(DFCI, Nat Med, 2018*), *(Broad, PNAS, 2012), (BCGSC, Blood, 2013*), *(TCGA, PanCancer Atlas), (Duke, Cell, 2017)*, ALL patients *(St Jude, Nat Genet, 2016), (St Jude, Nat Genet, 2015)*, and pediatric ALL patients (TARGET, 2018)—across public datasets in https://www.cbioportal.org. **B** U2OS cells were transfected with an empty or FLT4 expression plasmid along with a pg13 luciferase reporter vector or a luciferase reporter for p21 or MDM2 to determine p53 target genes activity. **C** U2OS cells were transfected with an empty or FLT4 expression vector for 24 h. The cells were then treated with 10 µg/mL of 5-FU for 6 h. The cell lysates were blotted for the indicated antibodies. **D** U2OS cells were transfected with different combinations of FLT4, MDM2, and MDMX plasmids along with pg13 luciferase reporter vector to determine endogenous p53 under genotoxic conditions with 5-FU. **E** HEK293T cells were transfected with various amounts of FLT4 expression plasmid for 24 h. The cell lysates were blotted for the indicated antibodies. **F** HEK293T cells were transfected with a combination of FLT4, MDMX and MDM2 expression plasmids for 24 h. The cell lysates were blotted for the indicated antibodies (LE low exposure, HE high exposure). **G** U2OS cells were transfected with a combination of FLT4, MDMX or MDM2 expression plasmids for 24 h. The cell lysates were blotted for the indicated antibodies. **H** U2OS cells were transfected with a combination of FLT4, MDMX, wild-type MDM2 or mutant MDM2 C464A expression plasmids for 24 h. The cell lysates were blotted for the indicated antibodies. **I** U2OS cells were transfected with a combination of FLT4, MDMX, and MDM2 expression plasmids and immunostained for MDMX (green) and MDM2 (red), while the nuclei were stained with Hoechst. Scale bar: 20 μm. *p < 0.05, ****p < 0.0001.

We first compared the frequency of TP53 mutations amongst various leukemia public cohorts [36–43] in the cBio Cancer Genomics Portal (http://cbioportal.org) (Fig. 1A). The incidence of TP53 mutations in the total number of patients was around 20% in Diffuse Large B-cell Lymphoma (DLBC) and less than 10% in ALL (pediatric or adult). Given the high wild-type status of TP53 in leukemia patients, we investigated whether FLT4 overexpression promotes tumorigenicity by suppressing p53 activity. To gain this general mechanistic insight, we used U2OS, a cell line harboring wild-type p53. We found a reduced p53 transcriptional activity in response to FLT4 overexpression using a p53-responsive luciferase reporter vector (Fig. 1B). Furthermore, U2OS cells were transfected with luciferase reporters, both of which have the p53-binding sites of the p21 and MDM2 promoters [44]. FLT4 overexpression had significantly decreased the transcriptional activity of p21 and MDM2 reporters (Fig. 1B). These results confirm the suppression of p53 transcriptional activity by FLT4 overexpression [13, 28]. We then confirmed that FLT4 overexpression reduces the p53 protein levels, under normal and genotoxic conditions (Fig. 1C).

Our observed p53 suppression suggests that FLT4’s mechanism may involve the key negative regulatory complex, MDMX and MDM2. We tested this hypothesis by transfecting various combinations of MDMX, MDM2, and FLT4 vector constructs and a p53 reporter vector in U2OS cells, followed by activating p53 through 5-Fluorouracil treatment (5-FU). As expected, we show the suppression of p53 reporter activity by separate MDMX and MDM2 transfections, which had less of an impact individually than their co-expression (Fig. 1D). Interestingly, FLT4 overexpression further influenced the combined MDMX/MDM2 suppression of p53 activity.

MDMX and MDM2 are well known to stabilize each other by forming a complex which consequently accumulates to bind and suppress available p53. After confirming FLT4’s role in p53 activity via MDM2/MDMX, we tested its effect on complex stability by transfecting FLT4 into HEK293T cells and assessing endogenous MDM2 and MDMX expression. Interestingly, FLT4 overexpression increased endogenous MDM2 and MDMX proteins in a concentration-dependent manner, correlating with an increasing amount of tyrosine phosphorylation (Fig. 1E). Similar results were obtained in U2OS cells (Fig. S1). To further validate this, we transfected different combinations of expression vectors encoding MDM2, MDMX, or FLT4 in HEK293T cells. Our data show that MDMX and MDM2 co-transfection increases MDM2 protein levels compared to transfection of MDM2 alone (Fig. 1F), while MDMX and MDM2 co-transfection with FLT4 further increased the amount of MDM2.

Given that FLT4 increases MDM2 levels, we proposed that this effect would decrease the amount of p53. While co-transfection of MDM2 and MDMX was able to decrease p53 protein levels compared to their individual transfections, FLT4 further exacerbated this reduction (Fig. 1G). To investigate whether p53 decrease was caused by increased ubiquitination of elevated MDM2, we analyzed p53 levels after transfection with an MDM2 mutant vector incapable of ubiquitination (MDM2 C464A) using U2OS cells [45]. Although FLT4 was able to stabilize MDM2 C464A with MDMX, it failed to decrease p53 protein levels when compared to its co-transfection with wild-type MDM2 and MDMX (Fig. 1H), implying the importance of the MDM2 ubiquitin ligase activity in FLT4-mediated decrease in p53.

Taken together, these results indicate that FLT4 overexpression promotes the stabilization of the MDM2/MDMX complex. This, in turn, increases the abundance of MDM2 available to degrade p53 via ubiquitination, reducing its transcriptional activity.

### FLT4 overexpression relocalizes the nuclear MDM2/MDMX complex to the cytoplasm

To study FLT4’s influence on the localization of MDMX and MDM2, we co-transfected various combinations of MDM2, MDMX and FLT4 encoding vectors in U2OS cells and determined the localization of MDM2 and MDMX by immunofluorescence. As previously shown, MDMX alone was found in the cytoplasm, whereas MDM2 alone was localized in the nucleus [46]. When MDMX and MDM2 were co-expressed, MDMX re-localized in the nucleus with MDM2 (Fig. 1I). However, when FLT4 was overexpressed, we detected the MDM2/MDMX complex in the cytoplasm, suggesting FLT4’s role in re-localizing the heterocomplex. Our immunofluorescence results also confirmed the protein stability from our Western Blot studies, where MDM2 is stabilized by MDMX and furthermore by FLT4. Taken together, these results show that FLT4 overexpression promotes the stability of the MDM2/MDMX complex and re-localizes it in the cytoplasm.

### FLT4 induces phosphorylation of MDMX on Ser-314 to promote MDM2/MDMX heterodimerization

**Fig. 2:**
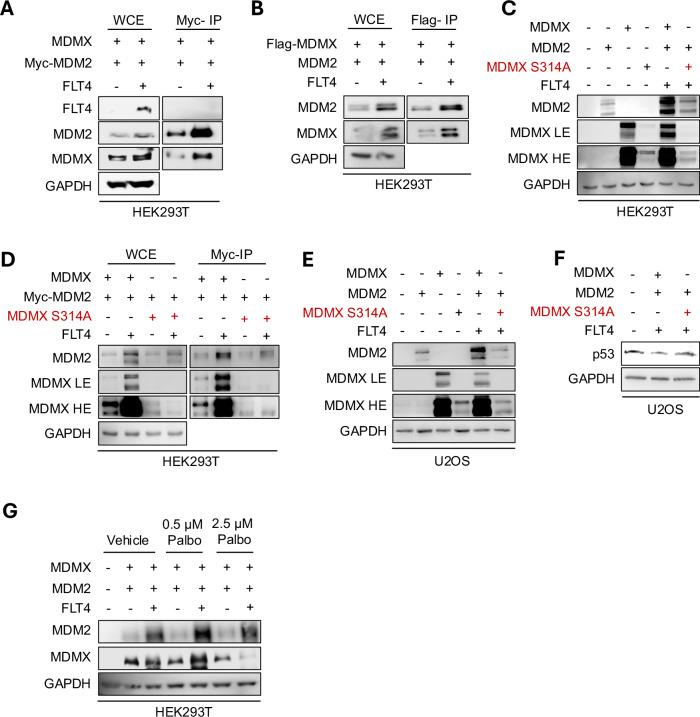
FLT4 activation leads to MDMX Ser-314 phosphorylation through the activation of CDK4/6 pathway. **A** HEK293T cells were transfected with a combination of MDMX, and Myc-MDM2 with or without FLT4 and harvested 24 h later for immunoprecipitation (IP) with Myc antibody-conjugated beads. Whole Cell Extract (WCE) and Myc IP samples were subjected to Western Blot analysis with the indicated antibodies. **B** HEK293T cells were transfected with a combination of Flag-MDMX, and MDM2 with or without FLT4 and harvested 24 h later for immunoprecipitation with Flag antibody-conjugated beads. Whole Cell Extract (WCE) and Flag IP samples were subjected to Western Blot analysis with the indicated antibodies. **C** HEK293T cells were transfected with various combinations of wild-type MDMX, mutant MDMX S314A, MDM2 and FLT4 plasmids. After 24 h of transfection, cells were harvested, and subjected to Western Blot analysis using the indicated antibodies. **D** HEK293T cells were transfected with either wild-type MDMX or mutant MDMX S314A in combination with Myc-MDM2 and FLT4 plasmids. After 24 h of transfection, cell lysates were harvested for IP with Myc-antibody-conjugated beads. WCE and Myc IP samples were subjected to Western Blot analysis with the indicated antibodies. **E**, **F** U2OS cells were transfected with various combinations of wild-type MDMX, mutant MDMX S314A, MDM2 and FLT4 plasmids. After 24 h of transfection, cells were harvested, and subjected to Western Blot analysis using the indicated antibodies. **G** HEK293T cells were transfected with a combination of MDM2, MDMX and FLT4 plasmids for 24 h, then treated with various concentrations of Palbociclib for 24 h. Cells were harvested and analyzed by Western Blot using the indicated antibodies. LE low exposure, HE high exposure.

We next investigated the molecular events leading to MDM2/MDMX stabilization by FLT4 overexpression. To validate the impact of FLT4 on MDMX and MDM2, HEK293T cells were transfected with Myc-MDM2 and Flag-MDMX with or without FLT4, then subjected to immunoprecipitation (IP) using Myc antibody-conjugated beads. As shown in Fig. 2A, the presence of FLT4 caused a greater pulldown of MDMX along with MDM2, suggesting a higher level of heterodimerization and subsequent stability of MDM2 (Fig. 2A). This result was validated with a reverse-IP using Flag-MDMX vector and Flag- antibody-conjugated beads (Fig. 2B). We have previously shown through Mass Spectrometry analysis (MS/MS) that Receptor Tyrosine Kinases can phosphorylate the same Ser-314 residue on MDMX [47]. Therefore, to test if FLT4 shares the same site of phosphorylation, we transfected HEK293T cells with FLT4 and MDM2 vectors, along with either wild-type MDMX or a mutant MDMX (MDMX S314A), wherein the Serine 314 residue was replaced with Alanine to prevent phosphorylation. Regardless of the MDMX S314A, MDM2, and FLT4 co-expression combination, we observed reduced stability of MDMX S314A compared to transfections with wild-type MDMX (Fig. 2C), impacting MDM2 levels accordingly. Furthermore, Myc immunoprecipitation of Myc-MDM2 revealed that Ser-314 phosphosite influenced the affinity between MDMX and MDM2 (Fig. 2D). This suggests that FLT4 signaling requires phosphorylation of Ser-314 on MDMX to increase the levels of available MDMX protein for binding with MDM2. We then assessed the impact of MDMX mutation in MDM2/MDMX and p53 modulation in U2OS cells. Our results show that MDMX S314A impaired the stabilization of both MDM2 and MDMX (Fig. 2E) and failed to decrease the levels of p53 under FLT4 activation (Fig. 2F).

To investigate the kinase responsible for MDMX phosphorylation, we analyzed the MDMX Ser-314 site using the kinase prediction software GPS. The highly stringent search criteria identified CDK4/6 as a kinase candidate. To verify this prediction, HEK293T cells were pretreated with Palbociclib, a specific inhibitor for CDK4/6, which impeded the ability of FLT4 to stabilize MDMX, as shown in Fig. 2G.

Therefore, our findings suggest that FLT4’s oncogenic effect involves phosphorylating MDMX at Ser-314 via CDK4/6. This stabilizes both MDMX and the MDM2/MDMX complex, ultimately leading to p53 inactivation.

### Activation of FLT4 in leukemic cells stabilizes the MDM2/MDMX complex to decrease p53

**Fig. 3:**
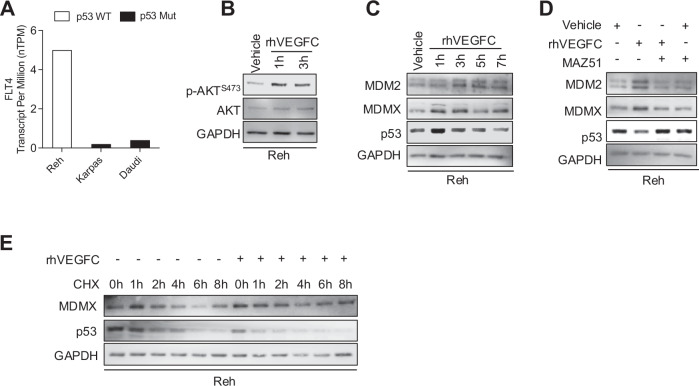
FLT4 stimulation with rhVEGFC stabilizes the MDM2/MDMX complex and decreases p53 in leukemic cells. **A** FLT4 expression in leukemic cell lines. Published RNA sequencing data of various in vitro ALL models were analyzed for FLT4 expression. The abundance in “Transcript Per Million” (TPM) was reported as the sum of the TPM values of all its protein-coding transcripts. The threshold level to detect the presence of a transcript for a particular gene was set to ≥1 TPM. **B**, **C** Reh cells were treated with 100 ng/mL of rhVEGFC for various time points. The cell lysates were blotted for the indicated antibodies. **D** Reh cells were pre-treated with FLT4 inhibitor, MAZ51 (5 μM), for 30 min, then treated with 100 ng/ml of rhVEGFC for 7 h. Cell lysates were collected for Western Blot analysis using the indicated antibodies. **E** Reh cells were pre-treated with 100 ng/mL of rhVEGFC for 3 h, then treated with 25 μg/mL of Cycloheximide (CHX) for various time points. The cell lysates were subjected to Western Blotting with the indicated antibodies.

We next examined the impact of stimulating endogenous FLT4 on the signaling mechanisms found in leukemia. Specifically, we used the ALL cell line Reh due to its expression of FLT4 and p53. Despite carrying a p53 with a heterozygous R181C mutation, Reh cells have a preserved p53 pathway in p21 and MDM2 transcriptional activity [48, 49], in contrast to well-known ALL cell lines that harbor inactivating p53 mutations (Figs. 3A and S2). To activate endogenous FLT4, we treated Reh leukemic cells with its ligand, rhVEGFC. FLT4 activity was measured through AKT phosphorylation and evaluated for its potential effect on the stability of intracellular MDMX and p53 levels. rhVEGFC was able to stimulate AKT phosphorylation (Fig. 3B), which corresponded to an increase in MDMX and a decrease in p53 levels (Fig. 3C). To confirm the rhVEGFC effect on the MDM2/MDMX complex, we treated the cells with FLT4’s specific inhibitor, MAZ51. A 30-min pre-treatment with MAZ51 reversed the rhVEGFC effect by decreasing MDMX and restoring p53 levels (Fig. 3D). In addition, to confirm the effect of FLT4 on the stability of MDMX and p53, we performed a treatment with CHX, a protein synthesis inhibitor. Our results show that FLT4 activation with its ligand increased the half-life of MDMX and reduced the stability of p53 (Fig. 3E).

### FLT4 modulates MDM2/MDMX and p53 to promote cell survival and chemotherapy resistance

**Fig. 4:**
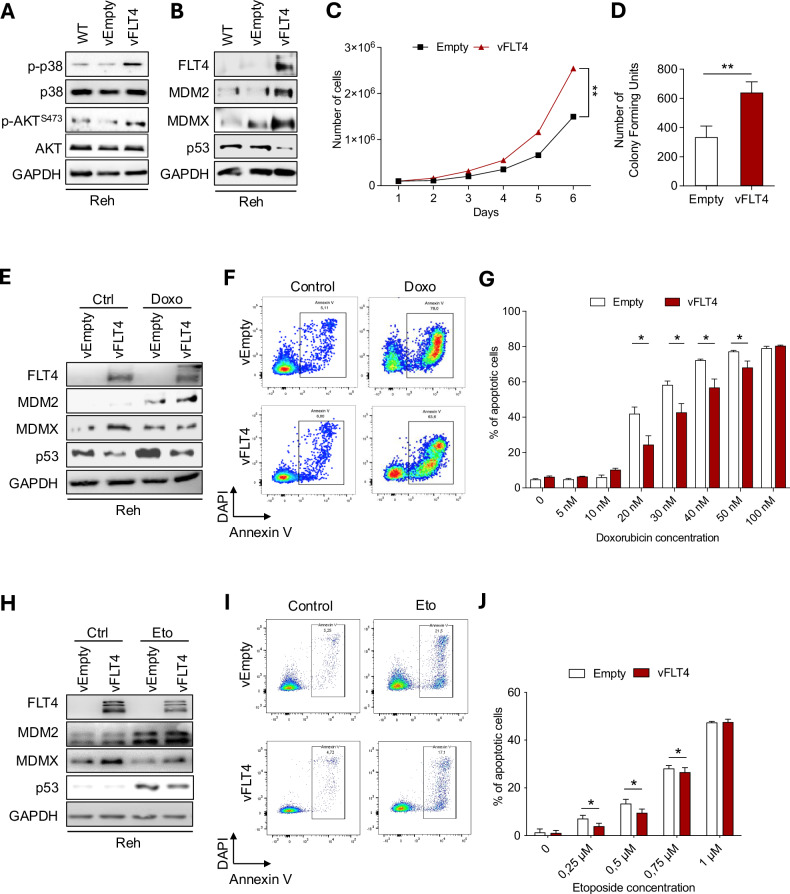
FLT4 overexpression decreases p53 promoting cell survival and resistance to chemotherapy. **A, B** Reh cells were transduced with FLT4 (vFLT4) or empty vector (vEmpty), then harvested and subjected to Western Blot with the indicated antibodies. **C** Reh cells transduced with FLT4 (vFLT4) or empty vector (vEmpty) were seeded in a six-well plate with a complete media and counted every day for five consecutive days. **D** Reh-transduced cells were plated in 1.2% methyl-cellulose. After 2 weeks of culture, colonies consisting of at least 50 cells were counted using an inverted microscope. **E** Reh-transduced cells were treated with Doxorubicin (50 nM, 5 h), then harvested and subjected to Western Blotting for the indicated antibodies. Reh-transduced cells were treated with 50 nM (**F**) or various amounts (**G**) of Doxorubicin for 48 h. Cells were stained with Annexin V and DAPI, and apoptosis was measured by flow cytometry. **H** Reh transduced cells were treated with Etoposide (0.25 μM, 3 h), then harvested and subjected to Western Blotting for the indicated antibodies. Reh-transduced cells were treated with 0.25 μM (**I**) or various amounts (**J**) of Etoposide for 24 h. Cells were stained with Annexin V and DAPI, and apoptosis was measured by flow cytometry. *p < 0.05, **p < 0.01.

To validate our results, we stably transduced FLT4 in Reh cells and found FLT4 overexpression to increase p38 and AKT phosphorylation, the protein levels of MDMX and MDM2 while decreasing that of p53 (Fig. 4A, B). Taken together, the influence of the FLT4 on MDM2/MDMX/p53 axis is conserved within an in vitro leukemic model.

Given FLT4’s ability to stabilize the MDM2/MDMX complex, we aimed to investigate its implication on p53-mediated cell death and response to DNA-damaging agents in leukemic cells. First, we observed that FLT4 overexpression in Reh increased cell proliferation (Fig. 4C) and colony formation (Fig. 4D). We next treated FLT4-overexpressing Reh cells with genotoxic drugs commonly used in the treatment of leukemia, Doxorubicin and Etoposide. The results showed FLT4-overexpressing cells having increased MDMX levels and lower levels of p53 in response to DNA damage by both genotoxic treatments (Fig. 4E, H). We then performed Annexin V staining to measure the amount of apoptosis under genotoxic conditions. Interestingly, FLT4-overexpressing cells showed lower levels of apoptosis compared to the controls with either Doxorubicin or Etoposide (Fig. 4F, I) in a dose-dependent manner (Fig. 4G, J). The same results on DNA damage response were obtained on adherent cells U2OS transfected with FLT4 and treated with 5-FU (Fig. S3). Collectively, these results show that FLT4 activation enhanced leukemic cell proliferation and reduced their sensitivity to DNA-damage-induced apoptosis, promoting their survival.

### Targeting CDK4/6 acts as a therapeutic strategy to overcome FLT4-induced chemoresistance in Leukemia

**Fig. 5:**
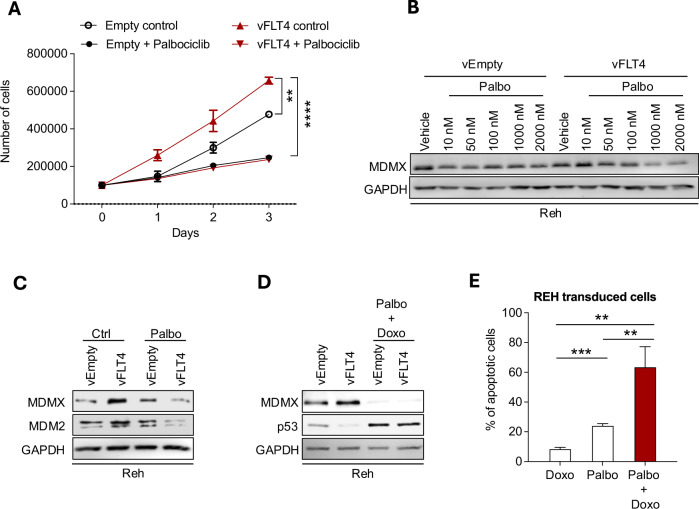
Inhibition of CDK4/6 prevents FLT4- induction of proliferation and -resistance to DNA damage and apoptosis. **A** Reh cells transduced with FLT4 (vFLT4), or empty vector (vEmpty) were seeded in a 6-well plate in complete media, then treated with the CDK4/6 inhibitor, Palbociclib (1 µM), at day 0 and counted for five consecutive days. **B** Reh cells transduced with FLT4 (vFLT4) or empty vector (vEmpty) were treated with CDK4/6 inhibitor, Palbociclib for various concentrations for 24 h. The cell lysates were subjected to Western Blotting with the indicated antibodies. **C** Reh transduced cells were pre-treated for 24 h with the CDK4/6 inhibitor, Palbociclib (1 μM), then harvested and subjected to Western Blotting using the indicated antibodies. **D** Reh transduced cells were pre-treated for 24 h with the CDK4/6 inhibitor, Palbociclib (1 μM) followed by 5 h of Doxorubicin treatment (50 nM), then harvested for Western Blotting using the indicated antibodies. **E** Reh transduced cells (vFLT4) were pre-treated with Palbociclib (1 μM) for 24 h and then treated with 50 nM of Doxorubicin for 48 h. Cells were stained with Annexin V and DAPI, and apoptosis was measured by flow cytometry. **p < 0.01, ***p < 0.001, ****p < 0.0001.

As our main finding, we observed that FLT4 influences the phosphorylation of the Ser-314 site on MDMX, through CDK4/6, which is partly responsible for the increased amount of the MDM2/MDMX complex and subsequent decrease in p53. As a therapeutic option, we tested blocking CDK4/6 to decrease the survival of FLT4-overexpressing leukemic cells and succumb them to DNA damaging therapy. First, we observed that the increased proliferation induced by FLT4 overexpression on Reh cells was significantly reduced by the pre-treatment with the CDK4/6 inhibitor, Palbociclib (Fig. 5A). CDK4/6 pathway inhibition with Palbociclib also reversed the effect of FLT4 overexpression on MDMX stability as shown in Reh cells treated with increasing concentrations of Palbociclib. (Fig. 5B). Furthermore, Palbociclib was able to inhibit the FLT4 effect on MDMX and p53 in normal condition (Fig. 5C) and genotoxic conditions (Fig. 5D). This results in a higher percentage of apoptosis in response to Doxorubicin in FLT4-overexpressing Reh cells (Fig. 5E). Similar results on MDMX and p53 were confirmed using another CDK4/6 inhibitor, Ribociclib (Fig. S4).

### FLT4 promotes leukemic cell growth in vivo

**Fig. 6:**
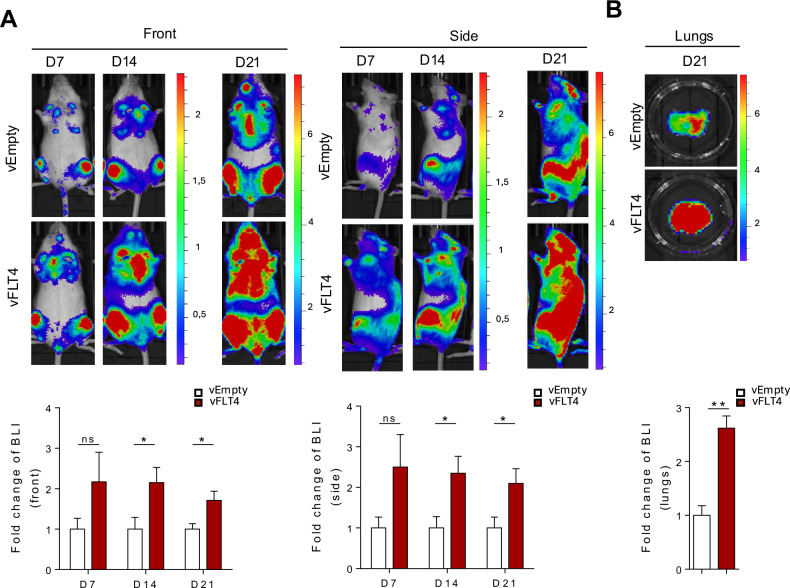
FLT4 activation increases leukemic cell proliferation in immunodeficient mice. **A** Reh cells transduced with FLT4 (vFLT4) or empty vector (vEmpty) were injected intravenously into the tail vein of female NOD/SCID mice. Whole body luminescence was detected by IVIS imaging shown by representative images on days 7, 14, and 21. **B** Lung tissues were isolated at day 21 and tumor growth was quantified by luminescence as shown by representative images. Each experiment was performed with an *n* = 5–7 per group. *p < 0.05, **p < 0.01.

We next examined the impact of FLT4 on leukemia progression in vivo by generating Reh cells expressing luciferase along with either FLT4 (vFLT4-Luc) or control (vEmpty-Luc) vectors. The luciferase activity between vectors was validated before injection (Fig. S5). Transduced Reh cells were injected intravenously into NOD SCID gamma immunodeficient mice and luciferase was activated by intraperitoneal injection at various time points (days 7, 14, and 21). Bioluminescence intensity was detected to quantify the tumor growth. Female mice exhibited increased cell growth in FLT4-overexpressing Reh cells (Fig. 6A), whereas male mice showed no difference (Fig. S6A), compared to their controls. Furthermore, we measured the luminescence signal in the lungs and we found that the female mice showed an increased signal in the FLT4-overexpressing Reh cells compared to their control (Fig. 6B), while no difference was observed in the males (Fig. S6B). Overall, these xenograft model results indicate that FLT4 accelerated tumor cell proliferation primarily in females.

## Discussion

Chemotherapy relies on p53 activation, crucial for responding to DNA damage in cancer cells. While solid tumors often have p53 mutations, leukemia mutations are rare (<10%). Inactivation, not mutations, may drive leukemic cell proliferation and resistance to chemotherapy, thus understanding chemoresistance mechanisms in leukemia is vital for improving treatments. Although targeting RTKs with inhibitors has demonstrated limited success in cancer treatment, their therapeutic impact on the p53 regulatory unit remains incompletely explored. Our findings established that FLT4 exhibits its oncogenic properties by influencing the CDK4/6 kinase, leading to an increase in the MDM2/MDMX heterocomplex and a reduction in p53 protein and DNA damage response.

RTK deregulation is common in several cancers, allowing tumor cells to increase their proliferation and survival [50, 51]. An understudied phenomenon is the overexpression of the RTK FLT4 and its specific ligand VEGFC, as well as its association with treatment failure, in pediatric acute lymphoid leukemia [28]. This suggests that the pro-survival pathway of FLT4 may be impeding the ability of p53 to suppress its tumor activity but also to respond to DNA-damaging therapies. However, to date, the FLT4 role in regulating p53 has not been studied. In this study, we provide evidence that the activation of FLT4 in leukemic cells, either through overexpression or rhVEGFC stimulation, stabilizes and relocates MDM2 and MDMX into the cytoplasm and reduces the levels of p53. Additionally, we found that the ubiquitination activity of MDM2 plays a crucial role in reducing p53 levels via FLT4. Since MDM2 and MDMX proteins are the main negative regulators of p53, these findings suggest that the cytoplasmic relocalization of MDM2/MDMX may be promoting the nuclear export of p53 for its ubiquitination-targeted degradation. Since p53 is a master tumor suppressor controlling cell cycle and proliferation, its degradation in leukemic cells correlated with higher survival under normal and genotoxic conditions, which resulted in greater colony size. Hence, we provide a mechanism through FLT4 that contributes to tumorigenesis and non-response to DNA damage by modulating the MDM2/MDMX/p53 axis.

The tumor suppressor protein p53 is a master regulator of the response to DNA damage. Upon cellular exposure to DNA damage, p53 stabilization leads to the transcriptional induction of genes involved in DNA repair, cell cycle arrest, apoptosis, and cellular senescence [10]. Our in vitro observations were translated in vivo through a xenograft model. FLT4 overexpression exhibited a consistent impact on leukemic cells in vivo actively promoting their proliferation. Furthermore, FLT4 increased the tumor burden of leukemic cells, as shown by their localization into the lungs, suggesting an important role of FLT4 in the regulation of leukemic cell extravasation. Interestingly, our results showed a sex-specific effect of FLT4 overexpression in tumor growth. Within the landscape of pediatric leukemia, emerging studies reveal a notable sex disparity with a predilection for higher incidence in males compared to females [52]. This observation will be investigated in future studies to gain a more comprehensive insight into the underlying mechanisms of the role of FLT4 in driving leukemia progression in males *versus* females.

To dissect the molecular mechanisms of p53 regulation by FLT4, we performed an MS/MS analysis on the MDM2/MDMX complex. Our results indicate that MDM2 plays a crucial role in regulating the stability of MDMX. Co-transfection of MDM2 and MDMX resulted in reduced levels of MDMX protein, suggesting that MDM2 may target MDMX for ubiquitination and subsequent proteasomal degradation. This regulatory mechanism underscores the dynamic interplay between MDM2 and MDMX, where MDM2’s ubiquitin ligase activity could govern the levels of MDMX, thereby influencing their complex formation and function in p53 regulation. Furthermore, we found that FLT4 increased MDMX phosphorylation at Ser-314, a site on the zinc finger domain of MDMX known to play a role in kinase signaling [12]. Previous studies on breast cancer and melanoma showed the significance of Ser-314 in regulating MDMX by RTK Her-4 and Axl [36, 49]. The importance of Ser-314 in our new model of FLT4 was confirmed using a mutant MDMX, where the Serine 314 site was replaced by Alanine, which led to a reduction in MDMX and a loss in the FLT4-induced stabilization of the heterodimer complex. One of the kinases predicted to be responsible for the phosphorylation of MDMX Ser-314 was CDK4/6, a master regulator of the cell cycle during the G1-S transition [53]. Certain cancers, such as hematological malignancies, exhibit an active CDK4/6 pathway alongside elevated MDM protein levels and wild-type p53. Inhibitors targeting CDK4/6 have shown efficacy in arresting the cell cycle progression of cancer cells. Our findings demonstrate that CDK4/6 inhibition reduces MDMX levels and enhances susceptibility to apoptosis in FLT4-overexpressing cells. This suggests that the MDMX zinc finger domain plays a crucial role in transmitting kinase signaling. Activation of CDK4/6 by FLT4 may lead to the phosphorylation of MDMX at Ser-314, inducing a conformational change that stabilizes MDMX. Following MDMX stabilization, it binds to and stabilizes MDM2, forming the MDM2/MDMX complex. This complex inhibits p53 tumor suppression and DNA-damaging response by sequestering p53 in the cytoplasm and degradation.

In conclusion, ALL harbor a wild-type p53 and often have elevated levels of FLT4/VEGFC, correlating with poor prognosis. In addition, rhVEGFC-treated leukemic cells induce mitogenic effects and protect against apoptosis. ALL may share a common mechanism of p53 suppression through the FLT4/VEGFC axis to support their carcinogenic effects and therapy resistance. For these reasons, our research proposes an innovative way to reactivate p53 through the pharmacological inhibition of FLT4 signaling as a novel treatment against ALL cell proliferation.

## Supplementary information

Supplementary figures

## Data Availability

All data generated or analyzed during this study are present in this published article.

## References

[1] Ferrando AA, López-Otín C. Clonal evolution in leukemia [Internet]. Nat Med. 2017;23:1135–45. 10.1038/nm.4410 28985206

[2] Maloney KW, Devidas M, Wang C, Mattano LA, Friedmann AM, Buckley P, et al. Outcome in children with standard-risk B-cell acute lymphoblastic leukemia: results of Children’s Oncology Group Trial AALL0331. 2019;38:602–12. 10.1200/JCO.19.01086 PMC7030893 31825704

[3] Whitehead TP, Metayer C, Wiemels JL, Singer AW, Miller MD. Childhood leukemia and primary prevention. Curr Prob Pediatr Ad. 2016;46:317–52. 10.1016/j.cppeds.2016.08.004 PMC5161115 27968954

[4] Schultz KR, Devidas M, Bowman WP, Aledo A, Slayton WB, Sather H, et al. Philadelphia chromosome-negative very high-risk acute lymphoblastic leukemia in children and adolescents: results from Children’s Oncology Group Study AALL0031. Leukemia. 2014;28:964–7. 24434862 10.1038/leu.2014.29 PMC4283793

[5] Nguyen K, Devidas M, Cheng SC, La M, Raetz EA, Carroll WL, et al. Factors influencing survival after relapse from acute lymphoblastic leukemia: a Children’s Oncology Group study. Leukemia. 2008;22:2142–50. 18818707 10.1038/leu.2008.251 PMC2872117

[6] Winick NJ, Carroll WL, Hunger SP. Childhood leukemia—new advances and challenges. N Engl J Med. 2004;351:601–3. 15295054 10.1056/NEJMe048154

[7] Oeffinger KC, Eshelman DA, Tomlinson GE, Buchanan GR, Foster BM. Grading of late effects in young adult survivors of childhood cancer followed in an ambulatory adult setting. Cancer. 2000;88:1687–95. 10738228

[8] Essig S, Li Q, Chen Y, Hitzler J, Leisenring W, Greenberg M, et al. Risk of late effects of treatment in children newly diagnosed with standard-risk acute lymphoblastic leukaemia: a report from the Childhood Cancer Survivor Study cohort. Lancet Oncol. 2014;15:841–51. 24954778 10.1016/S1470-2045(14)70265-7 PMC4142216

[9] Madhusoodhan PP, Carroll WL, Bhatla T. Progress and prospects in pediatric leukemia. Curr Prob Pediatr Ad. 2016;46:229–41. 10.1016/j.cppeds.2016.04.003 27283082

[10] Bieging KT, Mello SS, Attardi LD. Unravelling mechanisms of p53-mediated tumour suppression [Internet]. Nat. Rev. Cancer; 2014;14:359–70. 10.1038/nrc3711 PMC4049238 24739573

[11] Ashcroft M, Vousden KH. Regulation of p53 stability. Oncogene. 1999;18:7637–43. 10618703 10.1038/sj.onc.1203012

[12] Wade M, Li YC, Wahl GM. MDM2, MDMX and p53 in oncogenesis and cancer therapy. Nat Rev Cancer. 2013;13:83–96. 23303139 10.1038/nrc3430 PMC4161369

[13] Kruse JP, Gu W. Modes of p53 regulation. Cell. 2009;137:609–22. 10.1016/j.cell.2009.04.050 PMC3737742 19450511

[14] Wade M, Wang YV, Wahl GM. The p53 orchestra: Mdm2 and Mdmx set the tone. Trends Cell Biol. 2010;20:299–309. 10.1016/j.tcb.2010.01.009 PMC2910097 20172729

[15] Olivier M, Hollstein M, Hainaut P. TP53 mutations in human cancers: origins, consequences, and clinical use. Csh Perspect Biol. 2010;2:a001008. 10.1101/cshperspect.a001008 PMC2827900 20182602

[16] Bolouri H, Farrar JE, Triche T, Ries RE, Lim EL, Alonzo TA, et al. The molecular landscape of pediatric acute myeloid leukemia reveals recurrent structural alterations and age-specific mutational interactions. Nat Med. 2018;24:103–12. 29227476 10.1038/nm.4439 PMC5907936

[17] Quintás-Cardama A, Hu C, Qutub A, Qiu YH, Zhang X, Post SM, et al. p53 pathway dysfunction is highly prevalent in acute myeloid leukemia independent of TP53 mutational status. Leukemia. 2017;31:1296–305. 27885271 10.1038/leu.2016.350

[18] Hof J, Krentz S, Schewick C, van, Körner G, Shalapour S, Rhein P, et al. Mutations and deletions of the TP53 gene predict nonresponse to treatment and poor outcome in first relapse of childhood acute lymphoblastic leukemia. J Clin Oncol. 2011;29:3185–93. 21747090 10.1200/JCO.2011.34.8144

[19] Wada M, Bartram CR, Nakamura H, Hachiya M, Chen DL, Borenstein J, et al. Analysis of p53 mutations in a large series of lymphoid hematologic malignancies of childhood. Blood. 1993;82:3163–9. 8219205

[20] Carvajal LA, Neriah DB, Senecal A, Benard L, Thiruthuvanathan V, Yatsenko T, et al. Dual inhibition of MDMX and MDM2 as a therapeutic strategy in leukemia [Internet]. Sci Transl Med. 2018; p. 3003. 10.1126/scitranslmed.aao3003 PMC6130841 29643228

[21] Han X, Medeiros LJ, Zhang YH, You MJ, Andreeff M, Konopleva M, et al. High expression of human homologue of murine double minute 4 and the short splicing variant, HDM4-S, in bone marrow in patients with acute myeloid leukemia or myelodysplastic syndrome. Clin Lymphoma Myeloma Leuk. 2016;16:S30–8. 27155969 10.1016/j.clml.2016.03.012

[22] Bueso-Ramos C, Yang Y, deLeon E, McCown P, Stass S, Albitar M. The human MDM-2 oncogene is overexpressed in leukemias. Blood. 1993;82:2617–23. 8219216

[23] Gustafsson B, Stål O, Gustafsson B. Overexpression of MDM2 in acute childhood lymphoblastic leukemia. Pediatr Hemat Oncol. 1998;15:519–26. 10.3109/08880019809018313 9842645

[24] Han X, Garcia-Manero G, McDonnell TJ, Lozano G, Medeiros LJ, Xiao L, et al. HDM4 (HDMX) is widely expressed in adult pre-B acute lymphoblastic leukemia and is a potential therapeutic target. Mod Pathol. 2007;20:54–62. 17143258 10.1038/modpathol.3800727

[25] Kaindl U, Morak M, Portsmouth C, Mecklenbräuker A, Kauer M, Zeginigg M, et al. Blocking ETV6/RUNX1-induced MDM2 overexpression by Nutlin-3 reactivates p53 signaling in childhood leukemia. Leukemia. 2014;28:600–8. 24240203 10.1038/leu.2013.345 PMC3948158

[26] Zhou M, Gu L, Abshire T, Homans A, Billett A, Yeager A, et al. Incidence and prognostic significance of MDM2 oncoprotein overexpression in relapsed childhood acute lymphoblastic leukemia. Leukemia. 2000;14:61–7. 10637478 10.1038/sj.leu.2401619

[27] Zhou M, Yeager AM, Smith SD, Findley HW. Overexpression of the MDM2 gene by childhood acute lymphoblastic leukemia cells expressing the wild-type p53 gene. Blood. 1995;85:1608–14. 7888679

[28] de Jonge HJM, Weidenaar AC, ter Elst AC, Boezen HM, Scherpen FJG, et al. Endogenous vascular endothelial growth factor-C expression is associated with decreased drug responsiveness in childhood acute myeloid leukemia. Clin Cancer Res. 2008;14:924–30. 18245556 10.1158/1078-0432.CCR-07-1821

[29] de Jonge HJ, Valk PJ, Veeger NJ, ter Elst A, den Boer ML, Cloos J, et al. High VEGFC expression is associated with unique gene expression profiles and predicts adverse prognosis in pediatric and adult acute myeloid leukemia. Blood. 2010;116:1747–54. 20522712 10.1182/blood-2010-03-270991

[30] Dias S, Choy M, Alitalo K, Rafii S. Vascular endothelial growth factor (VEGF)–C signaling through FLT-4 (VEGFR-3) mediates leukemic cell proliferation, survival, and resistance to chemotherapy. Blood. 2002;99:2179–84. 11877295 10.1182/blood.v99.6.2179

[31] Nowicki M, Ostalska-Nowicka D, Kaczmarek E, Miskowiak B, Witt M. Vascular endothelial growth factor C—a potent risk factor in childhood acute lymphoblastic leukaemia: an immunocytochemical approach. Histopathology. 2006;49:170–7. 16879394 10.1111/j.1365-2559.2006.02465.x

[32] Uphoff CC, Drexler HG. Comparative PCR analysis for detection of mycoplasma infections in continuous cell lines. Vitr Cell Dev Biol - Anim. 2002;38:79–85. 10.1290/1071-2690(2002)038<0079:CPAFDO>2.0.CO;2 11928999

[33] Uphoff CC, Drexler HG. Detecting Mycoplasma contamination in cell cultures by polymerase chain reaction. Methods Mol Med. 2003;88:319–26. 10.1385/1-59259-406-9:319 14634244

[34] de Polo A, Luo Z, Gerarduzzi C, Chen X, Little JB, Yuan ZM. AXL receptor signalling suppresses p53 in melanoma through stabilization of the MDMX–MDM2 complex. J Mol Cell Biol. 2017;9:154–65. 27927748 10.1093/jmcb/mjw045 PMC5907837

[35] Calabrese V, Mallette FA, Deschênes-Simard X, Ramanathan S, Gagnon J, Moores A, et al. SOCS1 links cytokine signaling to p53 and senescence. Mol Cell. 2009;36:754–67. 20005840 10.1016/j.molcel.2009.09.044

[36] Chapuy B, Stewart C, Dunford AJ, Kim J, Kamburov A, Redd RA, et al. Molecular subtypes of diffuse large B cell lymphoma are associated with distinct pathogenic mechanisms and outcomes. Nat Med. 2018;24:679–90. 29713087 10.1038/s41591-018-0016-8 PMC6613387

[37] Lohr JG, Stojanov P, Lawrence MS, Auclair D, Chapuy B, Sougnez C, et al. Discovery and prioritization of somatic mutations in diffuse large B-cell lymphoma (DLBCL) by whole-exome sequencing. Proc Natl Acad Sci. 2012;109:3879–84. 22343534 10.1073/pnas.1121343109 PMC3309757

[38] Morin RD, Mungall K, Pleasance E, Mungall AJ, Goya R, Huff RD, et al. Mutational and structural analysis of diffuse large B-cell lymphoma using whole-genome sequencing. Blood. 2013;122:1256–65. 23699601 10.1182/blood-2013-02-483727 PMC3744992

[39] Hoadley KA, Yau C, Hinoue T, Wolf DM, Lazar AJ, Drill E, et al. Cell-of-origin patterns dominate the molecular classification of 10,000 tumors from 33 types of cancer. Cell. 2018;173:291–304.e6. 29625048 10.1016/j.cell.2018.03.022 PMC5957518

[40] Reddy A, Zhang J, Davis NS, Moffitt AB, Love CL, Waldrop A, et al. Genetic and functional drivers of diffuse large B cell lymphoma. Cell. 2017;171:481–94.e15. 28985567 10.1016/j.cell.2017.09.027 PMC5659841

[41] Zhang J, McCastlain K, Yoshihara H, Xu B, Chang Y, Churchman ML, et al. Deregulation of DUX4 and ERG in acute lymphoblastic leukemia. Nat Genet. 2016;48:1481–9. 27776115 10.1038/ng.3691 PMC5144107

[42] Andersson AK, Ma J, Wang J, Chen X, Gedman AL, Dang J, et al. The landscape of somatic mutations in infant MLL-rearranged acute lymphoblastic leukemias. Nat Genet. 2015;47:330–7. 25730765 10.1038/ng.3230 PMC4553269

[43] Alexander TB, Gu Z, Iacobucci I, Dickerson K, Choi JK, Xu B, et al. The genetic basis and cell of origin of mixed phenotype acute leukaemia. Nature. 2018;562:373–9. 30209392 10.1038/s41586-018-0436-0 PMC6195459

[44] Villiard É, Brinkmann H, Moiseeva O, Mallette FA, Ferbeyre G, Roy S. Urodele p53 tolerates amino acid changes found in p53 variants linked to human cancer. Bmc Evol Biol. 2007;7:180. 17903248 10.1186/1471-2148-7-180 PMC2072957

[45] Foo RSY, Chan LKW, Kitsis RN, Bennett MR. Ubiquitination and degradation of the anti-apoptotic protein ARC by MDM2*. J Biol Chem. 2007;282:5529–35. 17142834 10.1074/jbc.M609046200

[46] Shadfan M, Lopez-Pajares V, Yuan ZM. MDM2 and MDMX: alone and together in regulation of p53. Transl Cancer Res. 2012;1:88–9. 23002429 PMC3448287

[47] Gerarduzzi C, de Polo A, Liu XS, Kharbili ME, Little JB, et al. Human epidermal growth factor receptor 4 (Her4) suppresses p53 protein via targeting the MDMX-MDM2 protein complex implication of a novel MDMX SER-314 phosphosite*. J Biol Chem. 2016;291:25937–49. 27777309 10.1074/jbc.M116.752303 PMC5207067

[48] Carbonnier V, Leroy B, Rosenberg S, Soussi T. Comprehensive assessment of TP53 loss of function using multiple combinatorial mutagenesis libraries. Sci Rep. 2020;10:20368. 33230179 10.1038/s41598-020-74892-2 PMC7683535

[49] Kang JG, Lago CU, Lee JE, Park JH, Donnelly MP, Starost MF, et al. A mouse homolog of a human TP53 germline mutation reveals a lipolytic activity of p53. Cell Rep. 2020;30:783–92.e5. 31968253 10.1016/j.celrep.2019.12.074 PMC7021448

[50] Xu AM, Huang PH. Receptor tyrosine kinase coactivation networks in cancer. Cancer Res. 2010;70:3857–60. 20406984 10.1158/0008-5472.CAN-10-0163 PMC2875162

[51] Stommel JM, Kimmelman AC, Ying H, Nabioullin R, Ponugoti AH, Wiedemeyer R, et al. Coactivation of receptor tyrosine kinases affects the response of tumor cells to targeted therapies. Science 2007;318:287–90. 17872411 10.1126/science.1142946

[52] Garniasih D, Susanah S, Sribudiani Y, Hilmanto D. The incidence and mortality of childhood acute lymphoblastic leukemia in Indonesia: a systematic review and meta-analysis. PLoS ONE. 2022;17:e0269706. 35696384 10.1371/journal.pone.0269706 PMC9191700

[53] Otto T, Sicinski P. Cell cycle proteins as promising targets in cancer therapy. Nat Rev Cancer. 2017;17:93–115. 28127048 10.1038/nrc.2016.138 PMC5345933

