# Supplementary material for: FLT4 activation promotes acute lymphoid leukemia survival through stabilization of MDM2/MDMX and inactivation of p53
Source: Oncogenesis. 2025 May 2;14(1):14. doi: 10.1038/s41389-025-00552-7 (PMC12048674; doi:10.1038/s41389-025-00552-7)
Supplement: Supplementary file 1 — Supplementary figures [file 41389_2025_552_MOESM1_ESM.pdf]

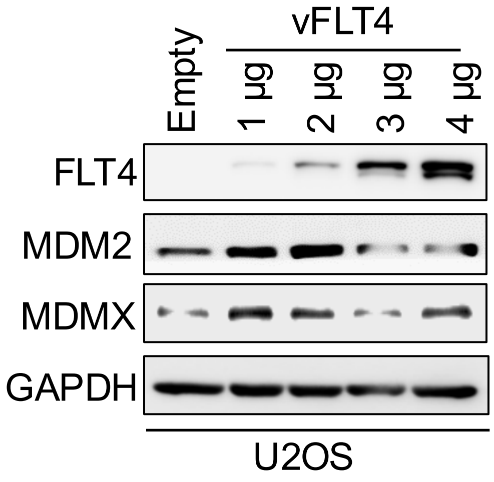

Figure S1: **FLT4 transfection increases the levels of MDM2/MDMX in a dose-dependent manner.** U2OS cells were transfected with various amounts of FLT4 expression plasmids for 24h. The cell lysates were blotted for the indicated antibodies.

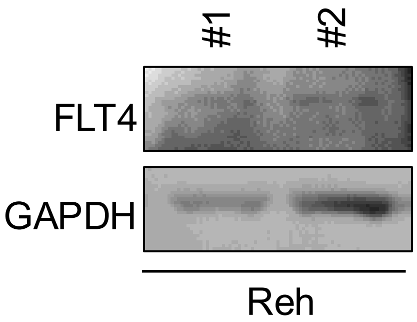

Figure S2: Reh cells express FLT4. Reh cells were harvested, and the cell lysates were subjected to blotting for the indicated antibodies.

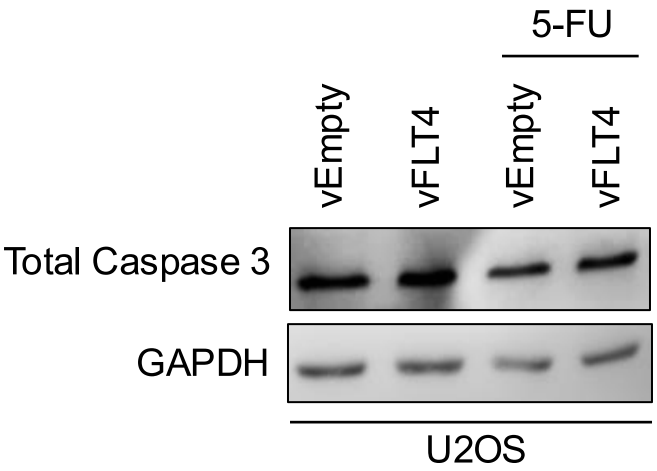

Figure S3: **FLT4 activation decreases the DNA damage response in adherent cells.** U2OS cells were transfected with various amounts of FLT4 expression plasmids for 24h, then treated with 1 μM of 5-FU for 6h. The cell lysates were blotted for the indicated antibodies.

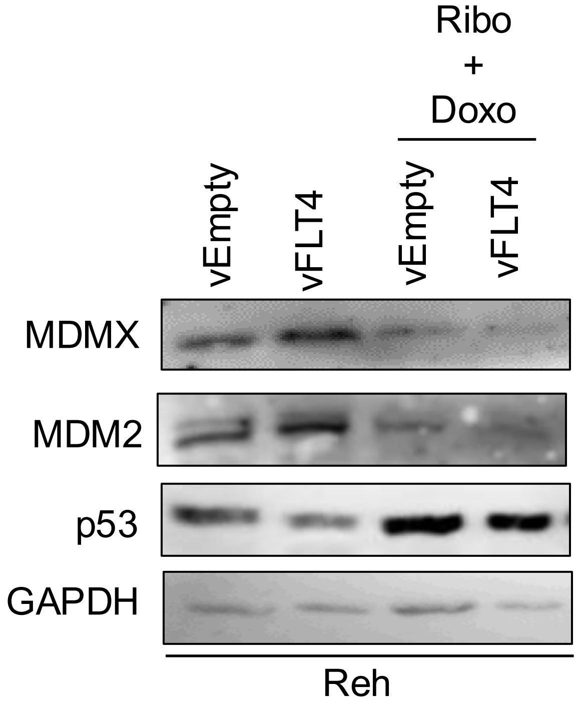

Figure S4: **Inhibition of CDK4/6 by Ribociclib prevents FLT4-induced stabilization of MDM2/MDMX and restores the levels of p53.** Reh transduced cells were pre-treated for 24h with the CDK4/6 inhibitor, Ribociclib (5 μM) followed by 5h of Doxorubicin treatment (50 nM), then harvested for Western Blotted using the indicated antibodies.

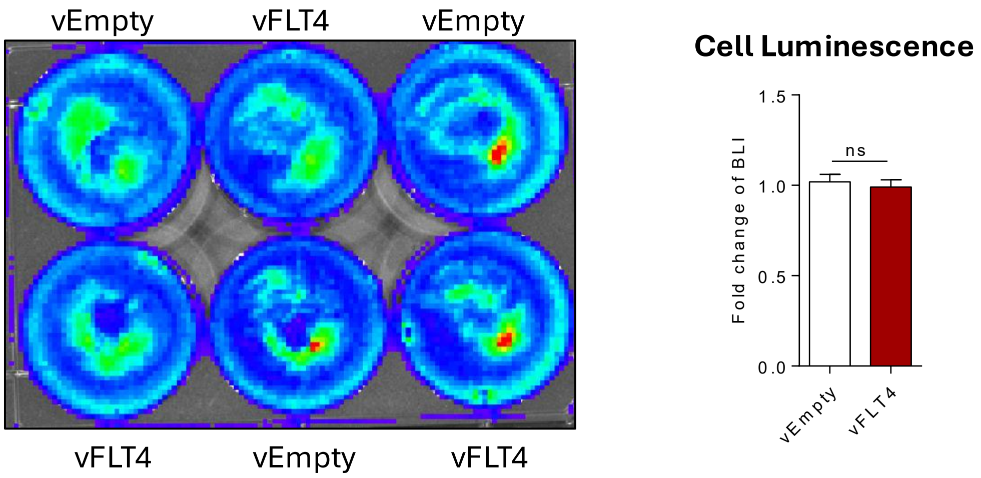

Figure S5: Reh transduced cells with vEmpty or vFLT4 express similar levels of Luciferase. Reh cells were first transduced with the dTomato-Luciferase vector. The dTomato-positive cells were sorted by flow cytometry and subjected to a second round of transduction with vEmpty-GFP or vFLT4-GFP. The GFP-positive cells from each condition were sorted by flow cytometry. Cells were plated at 500.000 cells/well, 150µg/mL of D-Luciferin was added directly to the media and after 5 minutes, the image was recorded. Bioluminescence Intensity (BLI) was detected by IVIS imaging system, and the signal difference was determined by t-test, ns: no significant difference.

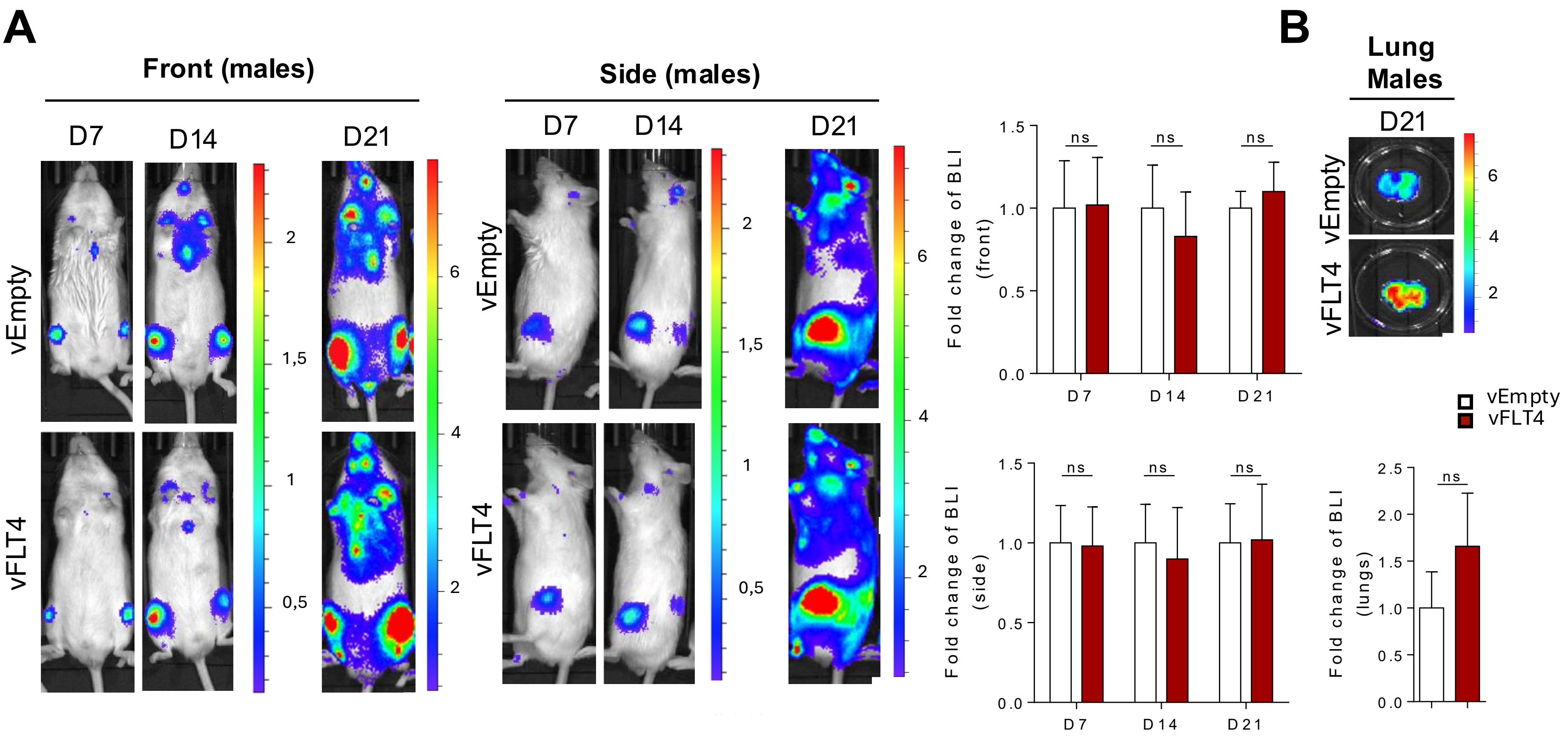

Figure S6: A) Reh cells transduced with FLT4 (vFLT4) or empty vector (vEmpty) were injected intravenously into the tail vein of male NOD/SCID mice. Whole body luminescence was detected by IVIS imaging shown by representative images on days 7, 14, and 21. **B)** Lung tissue was isolated at day 21 and tumor growth was quantified by luminescence as shown by representative images. Each experiment was performed with an n=5-7 per group. ns: no significant difference.
